# Autobiographical memory impairment in genetic generalized epilepsies: neurocognitive and pathophysiological determinants

**DOI:** 10.1055/s-0045-1804923

**Published:** 2025-03-25

**Authors:** Panayiotis Patrikelis, Eleni Loukopoulou, Elvira Masoura, Vasiliki Folia, Grigoris Kiosseoglou, Lambros Messinis, Sonia Malefaki, Giuliana Lucci, Vasileios Kimiskidis

**Affiliations:** 1 Aristotle University of Thessaloniki, School of Psychology, Laboratory of Neuropsychology and Behavioral Neuroscience, Thessaloniki, Greece.; 2 University Hospital of Patras, School of Medicine, Neuropsychology Section, Department of Psychiatry, Patras, Greece.; 3 University of Patras, School of Engineering, Department of Mechanical Engineering and Aeronautics, Patras, Greece.; 4 Università degli Studi Guglielmo Marconi, Dipartimento di Tecnologie, Comunicazione e Società, Roma, Italy.; 5 Aristotle University of Thessaloniki, School of Medicine, American Hellenic Educational Progressive Association (AHEPA) University Hospital, First Department of Neurology, Thessaloniki, Greece.

**Keywords:** Seizures, Memory, Episodic, Neuropsychology, Spatial Processing

## Abstract

**Background:**

The neuropsychological breakdowns of autobiographical memory (AM) in adults suffering from genetic generalized epilepsy (GGE) are far from being understood and largely neglected.

**Objective:**

We aimed at identifying AM impairments in GGE by analyzing neurocognitive deficits in illness-related variables possibly affecting AM.

**Methods:**

Patients with GGE were compared to healthy controls (HCs), through semistructured interviews on AM, as well as neuropsychological measures to identify potential determinants of AM impairment.

**Results:**

A single GGE group was formed by including patients with juvenile myoclonic epilepsy (JME), juvenile absence epilepsy (JAE), and epilepsy with generalized tonic-clonic seizures alone (EGTCA). Both GGE patients and HCs were tested for differential impairments in autobiographical episodic memory (AEM) and/or autobiographical semantic memory (ASEM), as well as other episodic- and/or semantic-memory and executive-function domains.

**Conclusion:**

The GGE patients exhibited overall impairment in autobiographical episodic and semantic information retrieval compared to HCs, both regarding childhood and the recent past. Furthermore, GGE patients demonstrated significantly poorer performance in immediate and delayed episodic recall, visuospatial working memory, visuoperceptual organization, face recognition memory, and verbal-executive functions compared to HCs. A distinct visuoperceptual involvement in retrieving childhood autobiographical episodic and semantic information has emerged, suggesting a potential connection between the latter AM systems and visual cognition.

## INTRODUCTION

The term *autobiographical memory* (AM) refers to a system encompassing memories encoded in the distant personal past. Traditionally, AM is defined as a form of episodic memory for personally-experienced events specific in time and place. 1 However, many researchers suggest that AM can be delineated into two intercorrelated but separate subsystems: autobiographical episodic memory (AEM) and autobiographical semantic memory (ASEM); 2 AEM pertains to specific and unique personal incidents, while ASEM relates to repeated and generalized personal facts. 2 Here are examples that may be of some help for the reader: AEM –“I remember the first day in Rome, when I missed the appointment with the house owner due to a flight delay and I called her from the metro station”; ASEM – “When I was fifteen, I spent Fridays practicing trumpet on specific military tracks and marching maneuvers for upcoming marching band performances”. The functional differentiation of AM appears to manifest at a neuroanatomical level as well. Studies investigating the neural underpinnings of AM indicate that AEM tends to involve the right medial temporal lobe, while ASEM seems to rely more on the left medial temporal lobe. 3

## Autobiographical memory in temporal lobe epilepsy patients

In addition to research focusing on healthy populations, the study of AM impairment could also be extended to patient populations, where it is as a significant cognitive comorbidity in various psychiatric 4 and neurological conditions. Notably, AM impairment features prominently in conditions such as epilepsy. 5 The neuropsychological exploration of memory function and dysfunction predominantly focused on temporal lobe epilepsy 6 (TLE), since the temporo-limbic system and its associated cognitive implications, rendering TLE a valuable tool for probing memory processes.

Several imaging, behavioral, and neuropsychological data support the role of the hippocampi in episodic memory. 7 It is well established that memory deficits in TLE are partly linked to epileptogenic lesions, such as hippocampal sclerosis, 8 as well as to the presence of a wider group of areas that are functionally abnormal in the interictal period (referred to as the *functional deficit zone* ). 9 Additionally, temporolimbic dysfunction in TLE has sparked debate among researchers regarding the relationship between epileptic activity and its effects on memory. 2 Therefore, TLE patients appear to be ideal candidates for the study of seizure-induced memory impairment, especially AM, due to the involvement of temporolimbic structures in memory and learning.

### Autobiographical memory in genetic generalized epilepsy patients

In contrast, AM study in other types of epilepsy has been largely neglected, particularly in the case of genetic generalized epilepsies (GGEs), which are highly-heritable conditions, possibly determined by polygenetic mechanisms, which account for 15% to 20% of all adult cases of epilepsy. 10 They comprise four key epilepsy syndromes: childhood absence epilepsy (CAE), juvenile absence epilepsy (JAE), juvenile myoclonic epilepsy (JME), and epilepsy with generalized tonic-clonic seizures alone (EGTCA), each exhibiting different electroclinical characteristics 11 and neuropsychological profiles 12 compared to focal epilepsies. This makes the study of remote memory (AM) in GGEs particularly intriguing, given the known effects of seizures on the consolidation of newly-acquired material in long-term memory. However, knowledge of whether primary generalized seizures per se link to deficits in the recall of AMs remains scarce. 13 The uniqueness of AEMs and their inability to be completely reestablished once compromised may render them more susceptible to disruption than ASEMs in view of the diffuse brain pathology and/or generalized seizures characterizing GGEs.

Compared to TLE patients, GGE patients show no morphological brain abnormalities on magnetic resonance imaging (MRI) scans and often do not display clinical signs between seizures. 14 Early neuropsychological studies into GGE suggested a primary impairment in sustaining and directing attention, 15 along with diminished performance in attention, memory, and psychomotor speed. 16 Moreover, dysexecutive manifestations have been observed in GGE patients. 17 18

To date, while cognitive profiling of GGEs has garnered attention, investigations into AM in adult GGE patients remain scarce, with pediatric patients receiving slightly more consideration. 13 . Given this gap, the present study aims to explore AM in adult GGE patients, specifically focusing on patients with JME, JAE and EGTCA. Moreover, we aim to assess the potential influence of seizures and disease-related factors on memory function, 13 in an attempt to discuss the putative role of cognitive pathophysiological mechanisms underlying declarative memory (DM) deficits in GGEs. Here, the term *cognitive pathophysiology* refers to the pathophysiology of cognitive disorders.

Before presenting our findings, we have considered important to provide the reader with a brief outline of neuropsychological impairments in GGEs, and how these impairments may affect AM.

### Neuropsychological differences among GGE subtypes

Furthermore, the question of whether cognition is differentially affected in GGE subtypes remains a matter of speculation. In relation to the CAE and EGTCA subtypes, there is a general consensus that JME patients exhibit characteristic features of cognitive impairment, which may be genetically determined and distinct from drug effects and seizure impacts. 19 Recent findings 20 point on the susceptibility of the auditory information processing in GGE patients (EGTCA and JME included) to the effects of idiopathic generalized seizures. Moreover, dysfunction in executive attention appears to be a salient neuropsychological feature in JME. 21 The EGTCA patients face an increased risk of developing psychiatric and neurocognitive comorbidities; 22 however the characterization of cognitive dysfunction patterns in this syndrome remains an ongoing area of research. 19

Additionally, there is evidence of visuospatial and language deficits in CAE and JAE, which are associated with atypical neurodevelopment. 19 Reduced right hippocampal volume has been linked to JAE, 23 while altered hippocampal structure and functional reorganization have also been observed in other GGE subtypes. 24 The increased state of brain excitability in the absence of seizures may contribute to interictal functional deactivation of the hippocampus and parahippocampal gyrus, possibly accounting for memory, reasoning, and executive deficits in JAE. 19 Finally, neuroimaging evidence 25 suggests that regions in the basal ganglia-thalamocortical network exhibit aberrant nodal centrality. As epilepsy progresses, reorganization of the caudate nucleus may aid in preventing seizure attacks, while changes in the hippocampus may serve to protect against cognitive impairments.

### Possible interactions regarding cognitive impairment in GGEs and AM

One might anticipate that AM performance could be linked to the aforementioned neuropsychological abnormalities of GGEs. This expectation arises from substantial evidence 26 highlighting the crucial role attention plays in the encoding of episodic memories, while internal attention (selection, modulation, and maintenance of internally-generated information) not only facilitates the retrieval of episodic memories but is also indispensable to render them conscious. When it comes to AM retrieval, voluntary retrieval activates more prefrontal regions compared to involuntary retrieval, 27 since the former necessitates a cyclic search process heavily reliant on executive control areas to regulate top-down processing. 28

A pertinent issue that requires clarification pertains to deficits in remote memory, which also encompasses AM, as a likely consequence of long-standing anterograde amnesia, wherein neurological damage impairs memory accumulation. However, there is evidence 29 suggesting that retrograde amnesia, which characterized by the loss of, or loss of access to, previously well-established memories, can also occur, albeit rarely, in the absence of any anterograde memory impairment. Reports 29 of neurological diseases causing such “focal retrograde amnesia” have generated considerable debate, an issue also relevant to GGEs.

## METHODS

### Participants

We used a case-control design for the present study. A convenience sample of 21 patients with GGE (JAE, EGTCA, and JME; with 10 female subjects) participated voluntarily. A unified GGE group was formed, comprising all three sub- syndromes (JME: n = 9; JAE: n = 6; and EGTCA: n = 7), with a mean age of 28.19(± 11.70) years and a mean of 12.33(± 2.39) years of schooling ( Table 1 ). In the total GGE sample, 5 patients were seizure free, and 9 were on monotherapy.

**Table 1:** Demographic and clinical characteristics

Clinical characteristics	GGE patients (n = 21)	HCs (n = 21)	Test statistic (U-test and t-test where appropriate)	*p* -value
Sex (female): n	10	9	0.096 ^a^	0.757
Age (years): mean ± SD	28.19(± 11.70)	32.05 (9.72)	−1.789	0.074
Years of schooling: mean ± SD	12.33(± 2.39)	13.52 (1.94)	−1.366	0.172
Age at epilepsy onset (years): mean ± SD	16.11(± 6.99)	N/A		
Yearly frequency of seizures: mean ± SD	2.25(± 1.48)	N/A		
Duration of epilepsy (years): mean ± SD	13.05(± 13.90)	N/A		
Seizure freedom (years): mean ± SD	3.43(± 4.60)	N/A		
Number of pills (ASMs): mean ± SD	1.56(± 0.89)	N/A		

Abbreviations: ASMs, antiseizure medications; GGE, genetic generalized epilepsy; HC, healthy controls; N/A, not available; SD, standard deviation.

Note: Seizure freedom is the time without occurrence of seizures.

The study was approved by the Ethics Committee of the First Department of Neurology of the American Hellenic Educational Progressive Association (AHEPA) University Hospital of Thessaloniki. All patients were seen at the Epilepsy Outpatient Clinic of a Tertiary Academic Hospital and provided informed consent for the procedures. The inclusion criteria were patients diagnosed by a consulting neurologist based on clinical and neurophysiological evidence, including MRI and video-electroencephalography (VEEG) monitoring. The exclusion criteria were history of brain injury, posttraumatic epilepsy, psychiatric and/or neurological disorders other than seizures, and mental retardation. Moreover, all patients underwent a comprehensive clinical interview to gather detailed seizure histories, including the precise onset and duration of seizures, the daily intake of antiseizure medications (ASMs), seizure frequency, and periods of seizure freedom.

### Control group

Healthy volunteers were invited by the research team to participate in the control group. The group of healthy controls (HCs) consisted of 21 participants (9 female subjects), matched demographically in terms of age, years of schooling, and gender. The HCs had an mean age of 32.05(± 9.72) years and a mean of 13.52(± 1.94) years of schooling. The gender distribution, mean age, and years of schooling did not significantly differ between the two groups ( Table 1 ).

The exclusion criteria for the control group included a history of psychiatric illness, head injury, and/or other neurological disorders. The HCs were screened by our expert neurologists after a clinical interview. All participants were native Greek speakers, predominantly residing in urban areas of Northern Greece.

### Materials

#### *Neuropsychological measure*

The neuropsychological measure implemented in this study are briefly described bellow.

#### *The Autobiographical Memory Interview 30*

The Autobiographical Memory Interview (AMI) is a semistructured interview schedule to assessing personal remote memory. The interview consists of two types of questions: personal semantic and autobiographical incidents, concurrently administered, that is, *autobiographical episodes embedded with semantic knowledge* . Thus, by conducting two separate interviews for both episodic and semantic memory, these latter AM memory components can be directly compared. In the episodic autobiographical interview, the interviewee is asked to provide descriptions of incidents across three broad periods: childhood (ages 0–18 years), early adulthood (ages 18–30 years), and the recent past (within the past 5 years), with each period having a maximum score of 21 points. Prompt questions raising the issue were asked about experiences with a friend or teacher during elementary and secondary school, first day of work at a job, wedding, a visit from a relative, a journey. The episodic richness and specificity of time and place can be then analyzed by the examiner. After that, the personal semantics interview aims at gathering general information about the personal past of the subject, that is, names of friends or work locations, from the three different periods. 30 It assesses the temporal gradient for episodic and personal semantic memory, irrespective of the life periods. The interview requires the examinee to recall memories of commonly-experienced events, rather than to respond to word cues. 30

The AMI was translated and adapted to the Greek language, and its administration and scoring procedures followed the guidelines of the AMI manual. Due to the relatively young age of the GGE patients and HCs, we decided to focus solely on two periods (childhood and the recent past) from the AMI. This choice was made due to the considerable overlap between early-adulthood and the recent past within our sample.

#### *Personal semantic questions*

The participants were required to recall facts from their past, such as home addresses, the locations of attended schools, and friends' names.

#### *Questions on autobiographical incidents*

The participants were asked to describe life incidents that occurred during each of the two periods. It is worth noting that we only included two out of the three AMI periods in our analysis. In their narratives, subjects were asked to provide specific temporal and spatial contextual information. For each period, three incidents were probed, such as “final school excursion”, for example. The participants' replies were recorded as verbatim as possible, and their memories were checked using information from medical files and reports from relatives.

All questions on autobiographical incidents were rated by two independent researchers, with the second being blinded to the subject group and the ratings given by the other researcher. The correlations between the scores provided by the 2 researchers for each period were *r* = 0.942 (childhood) and *r* = 0.948 (recent past). To test the authenticity and accuracy of the memories, relatives were asked to confirm the patients' recollections.

#### *Memory for stories*

We used the immediate story recall condition from the Logical Memory (LM) subtest of the Wechsler Memory Scale-Revised (WMS-R), 31 to assess immediate verbal episodic recall.

#### *Visual short-term/immediate memory, visuoperceptual ability, and visuospatial memory*

To assess visual recall, the Visual Patterns Test (VPT), 32 a measure of short-term nonverbal memory (visual working memory), was implemented given its relative independence of any spatial memory components.

The Rey-Osterrieth Complex Figure Test (RCFT) 33 assesses perceptual organization and visual memory. We used only the RCFT-copy section to assess visuoperceptual ability, and the RCFT delayed recall to assess visuospatial memory.

#### *Everyday memory*

Everyday memory was assessed through the Greek version of the Rivermead Behavioral Memory Test (RBMT), 31 which consists of several subtests that evaluate several memory functions, including immediate and delayed recall, recognition, and semantic and prospective memories. These subtests include RBMT name (delayed recall)/remembering people's names, RBMT story/remembering stories (immediate and delayed recall), RBMT pictures/remembering objects (delayed recognition of 10 out of 20 pictures), RBMT faces/remembering faces (delayed recognition of 5 out of 10 faces), RBMT personal item/remembering to collect personal belongings (delayed recall of the place where a subject's personal item has been hidden) RBMT bell (prospective memory)/scheduled actions, RBMT orientation/remembering a route (semantic memory, knowledge of personal or general facts), and RBMT date/remembering an appointment (semantic memory, general knowledge questions).

### Verbal executive functions and semantic memory

Verbal executive functions and semantic memory were evaluated through the phonemic verbal fluency (PVF) test, which assesses spontaneous word production starting with a given letter, while the semantic verbal fluency (SVF) variant entails the retrieval of words within a specific conceptual category (such as “animals”, for example). 34

### Procedure

The neuropsychological assessment of both groups was conducted in two separated sessions scheduled on the same day, with a break in between sessions.

### Statistical analysis

Initially, the data were examined visually for extreme outliers, that is, observations that are more extreme than the first quartile (Q _1_ ) - 3 x the interquartile range (IQR) or the third quartile (Q _3_ ) + 3 x the IQR), and all such data points were excluded from further analysis. Descriptive statistics were then computed for the clinico-demographic and neuropsychological variables. The assumption of normality of the data was tested using the Shapiro-Wilk test, since it is more powerful for small sample sizes (n < 50) than the commonly used Kolmogorov-Smirnov test. 35 When the normality hypothesis was rejected, the non-parametric Mann-Whitney U-test was used to examine differences between the two groups; otherwise, the standard *t* -test was used.

For cases in which the normality assumption was met, Hedge's g, which is a transformation of Cohen's d, was used as an effect size indicator. For cases in which the normality assumption was not met, Cliff's delta was initially estimated and then transformed to Cohen's d and Hedge's g to ensure the comparability of effect size indicators.

Moreover, correlation coefficients involving autobiographical memory, other neuropsychological measures, and clinical variables were computed.

All statistical tests were conducted at a 5% significance level, and the statistical analyses were performed using the IBM SPSS Statistics for Windows (IBM Corp., Armonk, NY, United States) software, version 27.0, and R language (R Foundation for Statistical Computing, Vienna, Austria) version 4.1.0.

## RESULTS

The demographic and clinical characteristics of the sample are presented in Table 1 . The GGE group, performed significantly worse compared to the controls in nearly all neuropsychological measures administered ( Table 2 ). Specifically, the former exhibited poorer performance in immediate (LM story Ire-WAIS, RBMT story-ImR) and delayed episodic recall (RBMT story-DRe), visuospatial working memory (VPT1), visuoperceptual organization (RCFT-copy), face recognition memory (RBMT faces), and verbal executive functions (SVF, SVF-clusters, PVF). Additionally, GGE patients showed impaired performance in both AEM (childhood and recent past) and ASEM (childhood and recent pest) periods compared to to HCs ( Table 3 ).

**Table 2:** Neuropsychological performance

		HCs	GGE	Test statistic (U-test and t-test where appropriate)	*p* -value	g
Neuropsychological measures						
LM story I Re-WMS-I	mean(± SD); (IQR)	18.29(± 3.84); (16.0)	14.90(± 3.56); (15.0)	2.960 (df = 39)	0.005	0.896
VPT1 visual	mean(± SD); (IQR)	23.33(± 2.82); (11.0)	17.75(± 4.09); (17.0)	5.113 (df = 39)	0.000	1.567
RCFT-copy	mean(± SD); (IQR)	35.90(± 0.44); (2.00)	33.62(± 6.52); (30.0)	305.5 (U)	0.004	0.596
RCFT-DRe	mean(± SD); (IQR)	21.14(± 6.24); (25.0)	17.57(± 7.17); (31.0)	1.721 (df = 40)	0.093	0.521
RBMT story-I ImRe	mean(± SD); (IQR)	19.14(± 3.32); (11.0)	12.12(± 3.36); (12.0)	309.0 (U)	0.000	2.128
RBMT story-I Dre	mean(± SD); (IQR)	8.90(± 0.30); (1.0)	8.56(± 0.51); (1.0)	225.5 (U)	0.019	0.512
RBMT pictures	mean(± SD); (IQR)	9.95(± 0.22); (1.0)	9.88(± 0.34); (1.0)	181.0 (U)	0.418	0.099
RBMT faces	mean(± SD); (IQR)	4.95(± 0.22); (1.0)	4.62(± 0.50); (1.0)	223.0 (U)	0.014	0.485
RBMT name	mean(± SD); (IQR)	3.71(± 0.72); (2.0)	3.25(± 1.24); (2.0)	198.0 (U)	0.206	0.241
RBMT personal items	mean(± SD); (IQR)	3.62(± 0.80); (2.0)	2.75(± 1.61); (4.0)	215.0 (U)	0.072	0.407
Semantic memory tests						
SVF	mean(± SD); (IQR)	51.86(± 5.47); (18.0)	37.50(± 8.52); (37.0)	395.0 (U)	0.000	2.444
SVF-clusters	mean(± SD); (IQR)	7.14(± 2.2); (9.0)	4.25(± 1.41); (5.0)	372.5 (U)	0.000	1.762
PVF	mean(± SD); (IQR)	35.00(± 8.33); (30.0)	22.77(± 6.81); (23.0)	4.960 (U)	0.000	1.561
PVF-clusters	mean(± SD); (IQR)	1.72(± 1.38); (5.0)	1.28(± 1.45); (5.0)	232.0 (U)	0.211	0.317

Abbreviations: df, degrees of freedom; Dre, delayed recall; GGE, genetic generalized epilepsy; HC, healthy control; ImRe, immediate recall; IQR, interquartile range; LM story I Re-WMS-I, Logical Memory story immediate recall-Weschsler Memory Scale-I; PVF, phonemic verbal fluency; RBMT story-I, RBMT-faces recall story I; RCFT, Rey-Osterrieth Complex Figure Test; SD, standard deviation; SVF, semantic verbal fluency; VPT, Visual Patterns test.

**Table 3:** Performance on the AMI

AMI measures		HCs	GGE	Test statistic (U-test)	*p* -value	g
AEM-childhood	mean(± SD); (IQR)	8.71(± 0.72); (3.0)	6.67(± 2.04); (7.0)	353.5	0.000	1.382
AEM-recent past	mean(± SD); (IQR)	8.57(± 0.68); (2.0)	6.45(± 1.67); (6.0)	370.0	0.000	1.705
ASEM-childhood	mean(± SD); (IQR)	20.74(± 0.62); (2.5)	18.77(± 1.72); (5.5)	366.5	0.000	1.629
ASEM-recent past	mean(± SD); (IQR)	20.48(± 0.98); (4.0)	19.22(± 1.45); (5.0)	330.5	0.001	1.035

Abbreviations: AEM, autobiographical episodic memory; AMI, Autobiographical Memory Interview; ASEM, autobiographical semantic episodic memory; GGE, genetic generalized epilepsy; HC, healthy control; IQR, interquartile range; SD, standard deviation.

No significant differences were found in terms of the performance in the AMI or other cognitive tests (OCTs) between GGE patients receiving mono- and polytherapy. Moreover, neither the age at onset nor the duration of epilepsy appeared to significantly affect the cognitive performance of GGE patients.

All correlation coefficients regarding epilepsy duration, epilepsy onset, AMI, and OCTs were weak, except for a significant negative correlation involving epilepsy duration and the measures of RCFT-DRe and VPT. Regarding the correlations concerning AMI measures and OCTs, AEM-childhood showed a significant positive correlation with RCFT-copy, while AMI-recent exhibited a significant negative correlation with RBMT-name. Moreover, ASEM-childhood demonstrated significant positive correlations with RCFT-copy and RBMT-pictures, while no significant correlations were identified for ASEM-recent.

## DISCUSSION

The present study aimed to investigate AM in a sample of adult GGE patients compared to HCs, and tested for differential impairments in AEM and/or ASEM, as well as in other domains, such as episodic, visual, and semantic memories, and executive function. We found that GGE patients experienced overall impairments in retrieving autobiographical episodic and semantic information for both periods compared to HCs.

Several studies have examined the impact of seizure-related variables on remote memory. Evidence 36 suggests that factors such as epilepsy duration and history of generalized seizures are unlikely to affect memory for autobiographical or public facts and events, a finding consistent with our results.

Furthermore, GGE patients exhibited differences from HCs in the domain of visuoperceptual organization (RCFT-copy). Of note, visuospatial impairments in JAE patients have been linked to atypical neurodevelopment 19 and reduced right hippocampal volume. 23

The diminished performance of GGE patients in the verbal executive domain (SVF, SVF-clusters, PVF) and visual working memory (WM; Visual Pattern Task, VPT) aligns with previous research highlighting executive function abnormalities in GGE, particularly in JME, in which frontal dysfunction has been documented. 21 37 Hopefully, these findings may serve to provide insights into the cognitive pathophysiology of GGEs.

In general, compared to HCs, the GGE group exhibited poorer performance in immediate and delayed episodic recall measures (LM story IRe-WMS-I, RBMT story-ImRe and Dre, Table 2 ), a finding partially consistent with recent findings. 20 Interestingly, interictal functional deactivation of the hippocampus and para-hippocampal gyrus in JAEs may contribute to deficits in memory, reasoning, and executive function. 25 38 Recent neuroimaging evidence 25 further suggests that the limbic system plays a role in abnormal resting-state functional networks in JAE. Similarly to the known effects of frontal lesions in episodic memory, 39 JME patients were expected to present with deficits in free episodic recall, given their well-documented presence of frontal lobe dysfunction. 24 37 Regarding GGEs, impaired face recognition memory has been observed, as evidenced by immediate face memory recognition problems in GEE patients. 40

However, it remains uncertain whether this finding represents a primary memory disorder or rather a secondary systemic manifestation due to primary seizure-related reticulo-thalamo-cortical (RTC) malfunction. This issue also applies to the present study, and a comparative investigation of the various subtypes within the GGE spectrum may clarify the underlying cognitive pathophysiological mechanisms. Notably, a greater vulnerability of the auditory information processing system to the effects of GGEs has been emphasized, 20 a fact linked to the administration modality of episodic memory tests.

Our findings regarding DM in adults with GGEs are in line with those of previous studies conducted in pediatric populations, 16 41 which have consistently demonstrated deficits in semantic memory, reduced performance in episodic memory, particularly in immediate and delayed episodic (story) recall, and attention deficits. Notably, the latter may partially account for GGEs' reduced episodic recall, as attention's pivotal role in memory performance, especially in encoding, has been emphasized. 42 Moreover, seizure-induced neuronal dysfunction may exacerbate semantic deficits by disrupting cognitive developmental trajectories, 8 therefore affecting semantic memory as well. Moreover, studies into the effects of ASMs suggest that chronic treatment may detrimentally influence cognitive development. 43

The ASEM deficit observed in our GGE patients aligns with previous and recent neuropsychological findings in adult JME patients, suggesting impairment in associative semantic processing. 17 19

The meta-analysis by Loughman et al. 44 provided evidence for consistent impairments in semantic knowledge and problem-solving skills, which recapitulates findings in mixed GGE samples. The latter exhibit reduced ability to manipulate acquired information, that is, semantic knowledge. Significantly lower scores in GGE compared to HCs were also found on measures such as the Wechsler Adult Intelligence Scale -WAIS Vocabulary and Information subtests, which gauge the extent of semantic knowledge and factual DM respectively. 44

Although DM has received considerable attention in the neuropsychological literature on GGEs, 13 the autobiographical components have been overlooked. The present study comparing GGE patients to HCs revealed impaired performance across two AEM and ASEM periods.

While delving into cognitive theories of memory exceeds the scope of the current study, it is noteworthy to mention that we did not observe the anticipated ASEM advantage in GGEs, as suggested by previous evidence for a double representation (double resistance) of semantic information. 45 This could account, in the case of the present study, for the construct of ASEM (knowledge about the self). 46 Moreover, since the GGE group exhibited impairments in the two AEM periods, the temporal gradient hypothesis (that is, temporally-graded retrograde memory loss with a disproportionate impairment of memory for events from the recent past relative to remote memories) did not find support.

Compared to ASEM, AEM presents a more complex and demanding system, as it requires control of the stored information, its temporal placing, processing, and confirmation via the prefrontal cortex: individuals must recall the elements of an event – time, place, and people – to mnemonically reconstruct the incident. 47 Memory recollections typically carry a significant emotional load, even when recalling and describing a specific episode. Autobiographical episodic memory includes various components, such as visual, verbal, emotional elements, whose activation involves a complex process requiring participation from visual associative areas, the amygdala, and frontal lobes. 48 In their AM model, Conway and Pleydell-Pearce 28 claim that AM disorders may arise either from diffuse cortical (occipital or frontotemporal) damage or disconnection. 28 The aforementioned findings are not surprising, as evidence suggests that generalized seizures may affect diverse regions, such as areas of the dominant temporal lobe and the frontal and parietal association cortices. 49

Positron-emission tomography (PET) studies on AM in healthy subjects put forward that autobiographical retrieval relies on conceptual knowledge, with components having representation in the bilateral temporal lobe. 50 The detrimental impact of generalized seizures on focal brain regions, coupled with the bilateral temporal distribution of AM representations, may account for AM deficits in GGE patients. Moreover, evidence suggests that in JAE, interictal limbic dysfunction may interfere with AM. The specific neurocognitive derailments characterizing EGTCA patients require further elucidation before advancing scientifically sound hypotheses regarding AM impairment.

Interestingly, our correlational data ( Table 4 ) suggest a selective involvement of visuoperceptual functions during the AEM and ASEM childhood periods. Positive correlations emerged with measures of visuoperceptual organization in AEM and ASEM regarding childhood, and with picture recognition exclusively for the latter. Early evidence suggests that individuals who report more subjectively vivid imagery on common questionnaires tend to recall past events and imagine future events with a greater number of visual and sensory details. 51

**Table 4:** Pearson correlations among AMI scores performance on and neuropsychological tests among GGE patients

	AEM-childhood	AEM-recent past	ASEM-childhood	ASEM-recent past
LM story IRe-WMS-I	−0.054	−0.165	0.176	−0.028
SVF	0.366	−0.046	0.335	0.065
SVF-clusters	−0.0016	−0.050	0.133	−0.042
PVF	0.121	0.128	−0.072	−0.338
PVF-clusters	0.008	−0.138	0.013	−0.165
RBMT story-I ImRe	0.133	0.184	0.064	0.223
RBMT pictures	0.194	−0.254	0.531*	−0.037
RBMT faces	0.216	−0.194	0.214	0.229
RBMT story-I Dre	0.187	−0.234	0.482*	0.062
RBMT name	0.076	−0.487*	0.076	0.473
RBMT personal items	−0.040	−0.082	0.089	0.394
RCFT-copy	0.555*	−0.188	0.462*	−0.224
RCFT-DRe	0.351	0.275	0.100	−0.047
VPT1 Visual	0.428*	0.141	0.194	−0.220

Abbreviations: AEM, autobiographical episodic memory; AMI, Autobiographical Memory Interview; ASEM, autobiographical semantic episodic memory; Dre, delayed recall; GGE, genetic generalized epilepsy; ImRe, immediate recall; LM story I Re-WMS-I, Logical Memory story immediate recall-Weschsler Memory Scale-I; PVF, phonemic verbal fluency; RBMT story-I, RBMT-faces recall story I; RCFT, Rey-Osterrieth Complex Figure Test; SVF, semantic verbal fluency; VPT, Visual Patterns test.

Note: *Correlation is significant at the 0.05 level (2-tailed).

Case studies involving patients with visual imagery impairment may provide valuable insights into potential shared brain networks supporting mental imagery and AM, despite being often anecdotal and complicated by multiple comorbidities. For instance, Ogden 52 reported a patient suffering from cortical blindness due to occipital infarctions, along with simultaneous visual imagery loss and autobiographical amnesia. Future studies in GGE patients should address the potential link implied here between early-life AM representations and visuospatial cognition.

Based on recent findings, 20 we herewith assume that the known effects of aberrant reticulo-thalamo-cortical dynamics inducing secondary-systemic cognitive impairment in GGEs may, among other already exposed factors, codetermine global AM deficits. The present study, to the best of our knowledge, is the first to focus on AM in adult GGE patients. The limitations to consider include the small size of the epilepsy subgroups, which may limit the generalization of findings, and the use of only one AM test. Additionally, due to the young age of the GGE sample, we excluded the early adulthood period from the study to prevent overlap between AEM and ASEM responses for adulthood and the recent past, which could represent a further limitation.

These findings warrant confirmation through future research on the cognitive pathophysiological mechanisms underlying GGE-related long-term memory impairments.

In conclusion, the current study showed that GGE patients (JME, EGTCA and JAE), exhibit global AM impairment. When compared to HCs, GGE patients displayed notable difficulties in recalling both episodic and semantic autobiographical information from childhood and the recent past. Additionally, GGE patients exhibited significantly lower performance in immediate and delayed episodic recall, visuospatial working memory, visuoperceptual organization, face recognition memory, and verbal executive functions. The challenges observed in both semantic and episodic components of AM in GGE patients may stem from secondary manifestations of primary cortical tone deregulation inherent in the pathophysiology of GGE, as shown elsewhere. 20 Furthermore, the diminished performance of patients in the verbal executive functions and visuospatial working memory domains may be suggestive of frontal lobe dysfunction, which is particularly prominent in JME. Finally, the study highlights a specific impairment in visuoperceptual functions related to retrieving autobiographical episodic and semantic information from childhood, possibly implying a rapport between early-life AM systems and visual cognition.
